# Presence of Infectious Bronchitis Virus Strain CK/CH/LDL/97I in the Middle East

**DOI:** 10.5402/2012/201721

**Published:** 2012-04-11

**Authors:** Mustafa Ababneh, Abd Elhafeed Dalab, Saad Alsaad, Mohammad Al-Zghoul

**Affiliations:** ^1^Faculty of Veterinary Medicine, Jordan University of Science and Technology, P. O. Box 3030, Irbid 22110, Jordan; ^2^Faculty of Veterinary Medicine and Animal Resources, King Faisal University, Al-Ihssa 31982, Saudi Arabia

## Abstract

Infectious bronchitis virus (IBV) is a very dynamic and evolving virus, causing major economic losses to the global poultry industry. In early 2011, respiratory disease outbreaks were investigated in Iraq, Jordan, and Saudi Arabia. Five IBV isolates (JOA2, JOA4, Saudi-1, Saudi-2, and Iraqi IBV) were detected by diagnostic-nested nucleocapsid RT-PCR. Strain identification was characterised by sequencing and phylogenetic analysis of the amplified hypervariable region of the spike 1 (S1) gene. These five IBV isolates were found to be of the IBV strain CK/CH/LDL/97I. Nucleotide identity between these five IBV isolates ranged from 96.9% to 99.7%, and between these isolates and the CK/CH/LDL/97I strain in the range of 96.6–99.1%. The sequenced fragment of the S1 gene of the CK/CH/LDL/97I strain had less than 80% nucleotide identity to the IBV vaccine strains commonly used in the Middle East (M41 and H120). The presence of these CK/CH/LDL/97I-like strains may account for vaccination failure against IBV, since all IBV isolates were from vaccinated chickens. In this paper, we documented for the first time the presence of IBV strain CK/CH/LDL/97I in the Middle East. This strain is known to have originated in China and Taiwan.

## 1. Introduction

Infectious bronchitis virus (IBV) is a positive sense single-stranded RNA virus, belonging to the genus *Coronavirus*, group 3 (gamma coronaviruses) [1]. Infectious bronchitis virus is not only an important pathogen of the respiratory system, but also can be nephropathogenic and cause infection of the reproductive system [2–4]. IBV was first recognised as avian respiratory pathogen in 1930, after that many IBV vaccines were introduced to tackle this problem (H52, H120, M41, 4/91(793/B), and other strains). Recently, different IBV variants have emerged causing nephropathogenic and reproductive problems which require a dramatic change in vaccination programmes [5, 6].

IBV strain CK/CH/LDL/97I was first reported in China in 1995 [7]. This strain was first implicated in proventriculitis in chickens [8], but in recent studies, this strain has been isolated from the trachea of infected chickens [9]. IBV CK/CH/LDL/97I strain accounts for 3.2% of total IBV strains found in China in the last 15 years [9]. The spike glycoprotein gene is the most variable gene in the IBV genome [10] and is composed of S1 and S2 subunits. Spike subunit 1 (S1) is about 1644 nt in length. The S1 protein is highly variable; it can differ from 20% to 25% and even up to 50% in the amino acid sequence among IBV serotypes [11]. This variability makes the S1 gene an ideal target in molecular assays to type IBV strains by RT-PCR and sequencing. The level of homology of the S1 subunit or part of it can predict cross-protection, that is, the higher the homology, the higher the chance of cross-protection [12, 13], but this rule is not always fulfilled [3]. Heterologous protection against the CK/CH/LDL/97I strain has not been achieved, since the nucleotide and amino acid homology of the CK/CH/LDL/97I S1 gene is only around 79% compared to the IBV vaccine strains. Only homologous protection has been achieved against CK/CH/LDL/97I [7].

In the Middle East, the common circulating IBV strains are Mass serotype vaccine isolates, H120 serotype vaccine isolates, D274 [14], IBV variant 1 strains (793/B, IS/222/96, IS/251/96, and IS/64714/96), and variant 2 strains (IS/223/96, IS/572/98, IS/585/98, and IS/589/98) [15], along with other unique strains like IS/885/00 [3] and the Egypt/Beni-Seuf/01 strain found in Egypt. By the end of 2009, variant 2 and variant 2-like strains appeared in Jordan (unpublished data) and in northern Iraq (Sul/01/09) [16].

In the early months of 2011 we noticed IBV outbreaks in spite of massive vaccination, this was due to the presence of a new strain of IBV. In this study, we documented the presence of the IBV strain CK/CH/LDL/97I for the first time in three Middle Eastern countries.

## 2. Materials and Methods

### 2.1. Tissue Samples and Viral RNA Extraction

Trachea, kidney, ovarian tissues, and cecal tonsils samples were collected from suspected IBV outbreaks in Iraq, Jordan, and Saudi Arabia. These samples were stored at −70°C in RNAlater solution (Qiagen, Germany) until RNA extraction was performed. Homogenised tissues were subjected to viral RNA extraction using the Viral Gene-spin Viral DNA/RNA extraction kit (Intron Biotechnology, Korea). Viral RNA extraction was performed according to the manufacturer's instructions. Viral RNA was stored at −70°C until analysis by RT-PCR. 

### 2.2. RT-PCR (Diagnostic and Phylogenetic)

The reverse-transcription (RT) step was performed using an RT system (Promega, USA). Briefly, viral RNA was denatured at 70°C for 10 minutes, followed by the addition of a reaction mix including 4 *μ*L MgCl_2_, 2 *μ*L reverse transcription 10x buffer, 2 *μ*L dNTP mixture (10 mM), 0.5 *μ*L random primers, 0.75 *μ*L AMV reverse transcriptase enzyme, 1 *μ*g RNA, and nuclease-free water to a final volume of 20 *μ*L. Then, the reaction was incubated at 42°C for 60 minutes, then 94°C for 5 minutes. The cDNA was diluted up to 100 *μ*L with nuclease-free water for PCR amplification. Two RT-PCR assays were used. First, a diagnostic-nested RT-PCR assay based on the amplification of the nucleocapsid (N) gene. IBV-specific oligonucleotides for the N gene were obtained according to a published primers sequences [17]; the IBV primers sequences are presented in Table 1. The diagnostic N gene amplification was done in a final volume of 25 *μ*L GoTaq Green master mix (Promega, USA) in which the final concentration of the N gene primers was 0.5 mmol. RT-PCR amplification was performed with a thermal profile of 94°C for 45 sec, 60°C (first step) and 53°C (second step) for 1 min and 72°C for 2 min for 40 cycles.

Next, a phylogenetic RT-PCR was performed using nested spike gene primers according to published protocols [4, 18]. The amplification profile was the same as that used for N gene-nested RT-PCR, but the annealing temperature for both RT-PCR steps was 50°C. Amplification was performed using the GenePro thermal cycler (Bioer, China). Nested S1 gene RT-PCR products were sequenced using an ABI Prism 310 genetic analyser at the Princess Haya Biotechnology Centre (Jordan University of Science and Technology, Jordan). Sequences were aligned using BioEdit (7.0.5.3) and MUSCLE (3.7) software. Aligned sequences were used for phylogenetic analysis. Maximum likelihood (ML) phylogenetic analysis with bootstrap values for *n* = 100 replicates was performed using the PhyML phylogenetic interface [19]. The nucleotide sequence identities were prepared using CLUSTAL W in the MEGALIGN Programme of the Lasergene software.

## 3. Results

Five IBV isolates were obtained in early 2011. Two IBV isolates were from Jordan (JOA2 and JOA4), two were from Saudi Arabia (Saudi-1 and Saudi-2), and one isolate was from Iraq (Iraqi IBV). The Jordanian isolates were isolated from layer farms at their peak of egg production, the Saudi strains were obtained from broilers and the Iraqi IBV isolate was obtained from a breeder farm as shown in Table 2. According to the clinical and gross examination coupled with the RT-PCR results from the different tissues (trachea, kidney, ovarian tissues, and cecal tonsils), these isolates had an extensive tissue tropism, different than the CK/CH/LDL/97I strain, which has tropism only for the respiratory system. The clinical signs ranged from respiratory to reproductive symptoms; two isolates (JOA2, Iraqi IBV) were implicated in kidney pathology in affected birds.

RT-PCR products of the diagnostic N gene assay were detected (380 bp) and for the phylogenetic S1 gene (392 bp). Direct-automated sequencing of the second RT-PCR product of nested S1 (SX3 and SX4 primers) was performed. The reference IBV strains included in the S1 sequence analysis from the United States were M41, Connecticut, DEL072, Ark/15C/96, and Beaudette. The European strains were 4/91, D3896, B1648, and H120. The Australian strains were N1/62 and N1/88. The Chinese strains were IBV LX4, QX IBV, J2, Q1, CK/CH/LDL/97I, and CK/CH/SCYA/10I. Middle Eastern IBV strains were IS/1201, IS/1366, IS/1464, IS/885, variant 1, variant 2, and Sul/01/09 as shown in Table 3.

All five IBV isolates (JOA2, JOA4, Saudi-1, Saudi-2, and Iraqi IBV) were found to be of the IBV strain CK/CH/LDL/97I and therefore they are CK/CH/LDL/97I-like strains. The CK/CH/LDL/97I strain is known to be endemic in China and Taiwan. The nucleotide identity between these five IBV isolates ranged from 96.9 to 99.7% with the Saudi-2 isolate being the most divergent. The identity of relatedness between the isolated IBV strains, CK/CH/LDL/97I, and the related strain CK/CH/SCYA/10I [20] is shown in Table 4. The Jordanian IBV isolate (JOA2) had a nucleotide identity of 98.1% and 99.4% to the CK/CH/LDL/97I and CK/CH/SCYA/10I strains, respectively. For the isolate JOA4, it had 98.8% and 99.7% nucleotide identity to the CK/CH/LDL/97I and CK/CH/SCYA/10I strains, respectively. The Saudi IBV isolates had a unique feature, in which isolated Saudi-1 shared 99.1% and 100% nucleotide identity to the CK/CH/LDL/97I and CK/CH/SCYA/10I strains, respectively, while the Saudi-2 had only 96.6% and 97.5% nucleotide identity to the CK/CH/LDL/97I and CK/CH/SCYA/10I strains, respectively. This could be due to an earlier introduction of this isolate to Saudi Arabia. The Iraqi IBV isolate shared 98.8% and 99.7% nucleotide identity to the CK/CH/LDL/97I and CK/CH/SCYA/10I strains, respectively.

**Figure 1:**
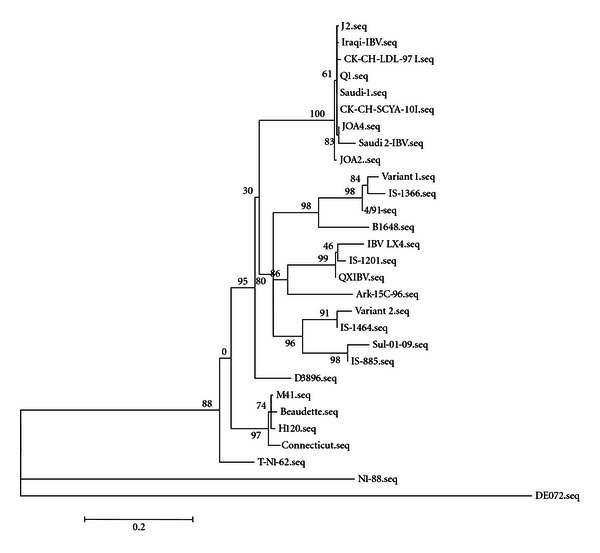
Phylogenetic tree for the five IBV isolates and related IBV strains based on the partial S1 sequence.

A phylogenetic tree was constructed based on the alignment of the partial S1 sequence. The five IBV isolates were aligned with other reference and related IBV strains as shown in Figure 1. It showed that all five IBV isolates were grouped with CK/CH/LDL/97I, CK/CH/SCYA/10I, J2, and Q1 (both J2 and Q1 were isolated from the proventriculus) [8] in one group. The nucleotide identity of these five isolates to the IBV vaccine strains in the three countries (M41, H120, and 4/91) were in the range of 78–82.1%, to variant 1 IBV strains in the range of 78.9–81.5%, to variant 2 IBV strains in the range of 81.7–83.6%, and to the IS/885 and Sul/01/09 strains in the range of 78.3–80.9% (data not shown).

Also, these IBV isolates represent a group distinct from the LX4 IBV type and from QXIBV and IS/1201 (a Middle Eastern strain) related to it. These strains share only 79% nucleotide sequence identity. The lowest nucleotide identity was seen with the DEL072 strain (58%) and with the N1/88 strains (63%) from Australia.

## 4. Discussion

In this study, the CK/CH/LDL/97I-like strains were isolated from three different Middle Eastern countries (Jordan, Saudi Arabia, and Iraq) in 2011. The CK/CH/LDL/97I-like strains were isolated from broilers in Saudi Arabia, layers in Jordan, and from breeders in Iraq. The CK/CH/LDL/97I-like strain was first isolated in China in 1995 [7]. This strain was isolated from the tracheas of infected chickens [9]. The IBV CK/CH/LDL/97I strains showed slight variability in the partial S1 sequence among IBV isolates from these three countries. The mode transmission of this strain from China to the Middle East is not clear; one reason might be that Chinese poultry vaccines and products have recently been registered and used in the Middle East.

All isolates came from flocks vaccinated with Mass-type H120, and 4/91 vaccines, which implies insufficient protection against these isolates. The poor relationship in the partial S1 sequence between the five IBV isolates and the vaccine strains (average nucleotide identity of 80%) could explain the failure of the Mass 41, H120, and 4/91 vaccination programmes to control IBV in these flocks [5]. There is no solution to tackle infection coming from these five IBV isolates except to make a homologous vaccine against this strain. Homologous vaccine was made for the IBV, CK/CH/LDL/97I strain found in China, for IBV QX strain and a nephropathogenic IBV strain in Korea [7, 21, 22].

In summary, this is the first paper indicting the presence of the IBV CK/CH/LDL/97I strain outside mainland China and Taiwan (in three Middle Eastern countries). In the near future, we expect that this strain might represent a serious problem for the poultry industry and there will be an urgent need to develop a homologous vaccine against this strain.

## Figures and Tables

**Table 1:** IBV primers for S1 and N genes used in this study.

IBV primer	Sequence 5′ to 3′	Position in S1 sequence	Reference
SX1	CACCTAGAGGTTTG T/C T A/T GCAT	677 to 698^A^	
SX2	TCCACCTCTATAAACACC C/T TT	1148 to 1168	[18]
SX3	TAATACTGG C/T AATTTTTCAGA	705 to 725	
SX4	AATACAGATTGCTTACAACCACC	1075 to 1097	

		Position in N sequence	

N784	AATTTTGGTGATGACAAGATGA	763 to 784^B^	
N1145	CATTGTTCCTCTCCTCATCTG	1145 to 1165	[17]
N791	GTGATGACAAGATGAATGAGGA	770 to 791	
N1129	CAGCTGAGGTCAATGCTTTATC	1129 to 1150	

^A^Nucleotide position according to IBV strain 793/B, accession number Z83979.

^B^Nucleotide position according to IBV strain Beaudette, accession number M95169.

**Table 2:** IBV isolates with their tissue tropism (according to the presence of IBV by diagnostic N gene RT-PCR) and clinical history.

Isolate	Tropism	Clinical findings	Vaccine used	Chicken/age
JOA4	Trachea, ovary	Drop in egg production	H120	
		Watery cysts in ovaries	M41	Layer, 24 weeks
		Short and enlarged oviducts	4/91	

JOA2	Trachea, kidney, ovary	Drop in egg production	H120, M41, 4/91	Layer, 22 weeks

Saudi-1	Trachea	Respiratory signs	H120, 4/91	Broiler, 22 days

Saudi-2	Trachea	Respiratory signs	H120, 4/91	Broiler, 24 days

Iraqi IBV	Trachea, kidney	Respiratory signs, nephritis, and drop in egg production	H120, M41, 4/91	Breeder, 25 weeks

**Table 3:** IBV strains with GenBank accession numbers used in this study.

IBV strain	Tropism	GenBank Accession No.
M41	Respiratory	M21883
Connecticut	Respiratory	L18990
DEL072	Respiratory	U77298
Ark/15C/96	Respiratory	AF169859
Beaudette	Respiratory	X02342
4/91	Respiratory	AF093794
D3896	Respiratory	X52084
B1648	Nephropathogenic	X87238
H120	Vaccine	M21970
LX4	Proventriculus/nephropathogenic	HM194716
QXIBV	Proventriculus	AF193423
CK/CH/LDL/97I	Respiratory	EF030998
CK/CH/SCYA/10I	Nephropathogenic	HM363027
J2	Proventriculus	AF286303
Q1	Proventriculus	AF286302
IS/1201	Respiratory	DQ400359
IS/1366	Respiratory/nephropathogenic	EU350550
IS/1464	Respiratory/nephropathogenic	EU780077
IS/885	Nephropathogenic	AY279533
Variant 1	Respiratory/nephropathogenic	AF093795
Variant 2	Respiratory/nephropathogenic	AF093796
Sul/01/09	Respiratory/nephropathogenic	GQ281656
N1/62	Nephropathogenic	U29522
N1/88	Respiratory	U29450

**Table 4:** The identity nucleotide identity of partial S1 among the five IBV isolates compared to the CK/CH/LDL/97I and CK/CH/SCYA/10I strains.

IBV strain/isolate	JOA2	JOA4	Saudi-1	Saudi-2	Iraqi IBV	CK/CH/LDL/97I	CK/CH/SCYA/10I
JOA2	100						
JOA4	99.1	100					
Saudi-1	99.4	99.7	100				
Saudi-2	96.9	97.5	97.5	100			
Iraqi IBV	99.1	99.4	99.7	97.2	100		
CK/CH/LDL/97I	98.1	98.8	99.1	96.6	98.8	100	
CK/CH/SCYA/10I	99.4	99.7	100	97.5	99.7	99.5	100
